# Effects of a wearable exoskeleton stride management assist system (SMA®) on spatiotemporal gait characteristics in individuals after stroke: a randomized controlled trial

**DOI:** 10.1186/s12984-015-0062-0

**Published:** 2015-08-20

**Authors:** Carolyn Buesing, Gabriela Fisch, Megan O’Donnell, Ida Shahidi, Lauren Thomas, Chaithanya K. Mummidisetty, Kenton J. Williams, Hideaki Takahashi, William Zev Rymer, Arun Jayaraman

**Affiliations:** Northwestern University Physical Therapy and Human Movement Sciences, 645 N. Michigan Ave, Suite 1100, Chicago, IL 60611 USA; Max Nader Lab for Rehabilitation Technologies and Outcomes Research, Rehabilitation Institute of Chicago, 345 E. Superior St, Chicago, IL 60611 USA; Honda R&D Americas, Inc, 21001 State Route 739, Raymond, OH 43067 USA; Director, Research Planning, Rehabilitation Institute of Chicago, 345 E. Superior St, Chicago, IL 60611 USA

## Abstract

**Background:**

Robots offer an alternative, potentially advantageous method of providing repetitive, high-dosage, and high-intensity training to address the gait impairments caused by stroke. In this study, we compared the effects of the Stride Management Assist (SMA®) System, a new wearable robotic device developed by Honda R&D Corporation, Japan, with functional task specific training (FTST) on spatiotemporal gait parameters in stroke survivors.

**Methods:**

A single blinded randomized control trial was performed to assess the effect of FTST and task-specific walking training with the SMA® device on spatiotemporal gait parameters. Participants (*n* = 50) were randomly assigned to FTST or SMA. Subjects in both groups received training 3 times per week for 6–8 weeks for a maximum of 18 training sessions. The GAITRite® system was used to collect data on subjects’ spatiotemporal gait characteristics before training (baseline), at mid-training, post-training, and at a 3-month follow-up.

**Results:**

After training, significant improvements in gait parameters were observed in both training groups compared to baseline, including an increase in velocity and cadence, a decrease in swing time on the impaired side, a decrease in double support time, an increase in stride length on impaired and non-impaired sides, and an increase in step length on impaired and non-impaired sides. No significant differences were observed between training groups; except for SMA group, step length on the impaired side increased significantly during self-selected walking speed trials and spatial asymmetry decreased significantly during fast-velocity walking trials.

**Conclusions:**

SMA and FTST interventions provided similar, significant improvements in spatiotemporal gait parameters; however, the SMA group showed additional improvements across more parameters at various time points. These results indicate that the SMA® device could be a useful therapeutic tool to improve spatiotemporal parameters and contribute to improved functional mobility in stroke survivors. Further research is needed to determine the feasibility of using this device in a home setting vs a clinic setting, and whether such home use provides continued benefits.

**Trial registration:**

This study is registered under the title “Development of walk assist device to improve community ambulation” and can be located in clinicaltrials.gov with the study identifier: NCT01994395.

## Introduction

Stroke is the leading cause of adult-onset disability. Recent studies estimate that stroke affects about 795,000 people in the U.S. each year, resulting in a prevalence of over 6.4 million stroke survivors [1, 2]. In the U.S., stroke results in an estimated annual cost of $53.9 billion, of which $36.5 billion reflects direct healthcare costs and the remainder is due to loss of productivity [3]. Recent statistics project an exponential increase in the global burden of stroke in the decades to come, particularly in low and middle-income countries [4]. With the high prevalence of stroke and costly demands of care, determining the most effective and efficient methods for stroke rehabilitation is critically important to reduce the overall burden stroke places on the healthcare system and on individual lives.

The goal of post-stroke rehabilitation is to reintegrate individuals back to their highest level of function for employment and social and community participation [5]. A large proportion of stroke survivors (up to 80 %) experience considerable gait deficits, limiting their capacity for community ambulation [6]. Studies have shown that after stroke, individuals demonstrate changes in two important gait parameters, velocity and symmetry. Velocity is known to decrease, while spatial and temporal gait parameters show pronounced asymmetries. Gait velocity following stroke has been found to range from 18 to 103 cm/s [7–10], whereas the average for healthy adults is 140 cm/s [11]. More than 50 % of individuals with chronic disability after stroke are known to exhibit temporal and spatial gait asymmetries [12]. Typical asymmetry characteristics after stroke include larger swing time / smaller swing time and/or larger stance time/ smaller stance time (i.e., temporal asymmetry) [12–14] and a larger step length /smaller step length (i.e., spatial asymmetry) [15].

A commonly expressed goal of stroke survivors is to ambulate with a more normal gait pattern and increased gait velocity [16]. In order to address this goal, gait training is typically a major part of the rehabilitation process. Several studies provide evidence of significant progress in gait velocity through physical therapy [6, 17–20]; however, there is limited evidence for significant improvements in spatial and temporal asymmetries (i.e. cadence, step time, step length, stride length, swing time, stance time, and double support time) following the rehabilitation process [21]. Gait asymmetry may have other long-term health consequences due to the increased demand placed on the non-paretic limb. Bringing individuals closer to a symmetrical gait pattern could improve energy efficiency, gait speed, and balance control, in addition to decreasing the risk of falls, lower extremity musculoskeletal injury, and loss of bone mineral density in the paretic limb [12, 21, 22].

Various methods and outcome measures have been used to assess an individual’s gait characteristics, such as picture video systems, Force Sensitive Resistor (FSR)–based pressure mats, and force platforms [23]. The GAITRite® system is a computerized assessment tool that utilizes an electronic walkway mat consisting of pressure-sensitive pads. GAITRite software recreates the steps an individual takes as they walk across the mat and calculates several spatiotemporal parameters, such as step length, swing time, and velocity. Use of the GAITRite system in assessing individuals with stroke has been shown to have strong inter- and intra-rater reliability [24], in addition to good test-retest reliability when assessing spatiotemporal parameters of gait, with an intraclass correlation coefficient (ICC) in the range of 0.69-0.99 [22, 25, 26]. As improving gait is one of the main rehabilitation goals after stroke, an accurate and reliable tool such as the GAITRite to assess gait characteristics is vital in evaluating the effectiveness of different treatment methods.

Recovery of gait function after stroke is thought to be driven by neural plasticity, which refers to changes in neuronal organization that allow recovery and functional adaptations after an insult to the brain [27]. In order to encourage neural plasticity, an individual needs to be provided with experiences and practice that allow learning and sensory input [28, 29]. Research has shown that current rehabilitation strategies can provide these experiences through high repetition, high intensity, and task-oriented movements [27, 30–34]. Within the last decade, an increasing amount of research and development has focused on the use of robotics for post-stroke rehabilitation. Robots can readily provide repetitive, high-dosage, and high-intensity training [27, 35], while reducing the labor and manual burden on therapists during the rehabilitation process [27]. Specifically, in individuals with stroke, two different types of robotic devices, end-effector and exoskeleton robots, can effectively complement conventional physical therapy to improve gait function [36]. The newly developing field of wearable robotics has the potential to provide additional advantages such as being easily transportable, more natural to use, and simple to control [37]. Wearable robots could be also used at home as a therapeutic technology both for assisting individuals with disabilities to perform activities of daily living and a means to continue rehabilitation outside of a formal clinical setting [38].

**Fig. 1:**
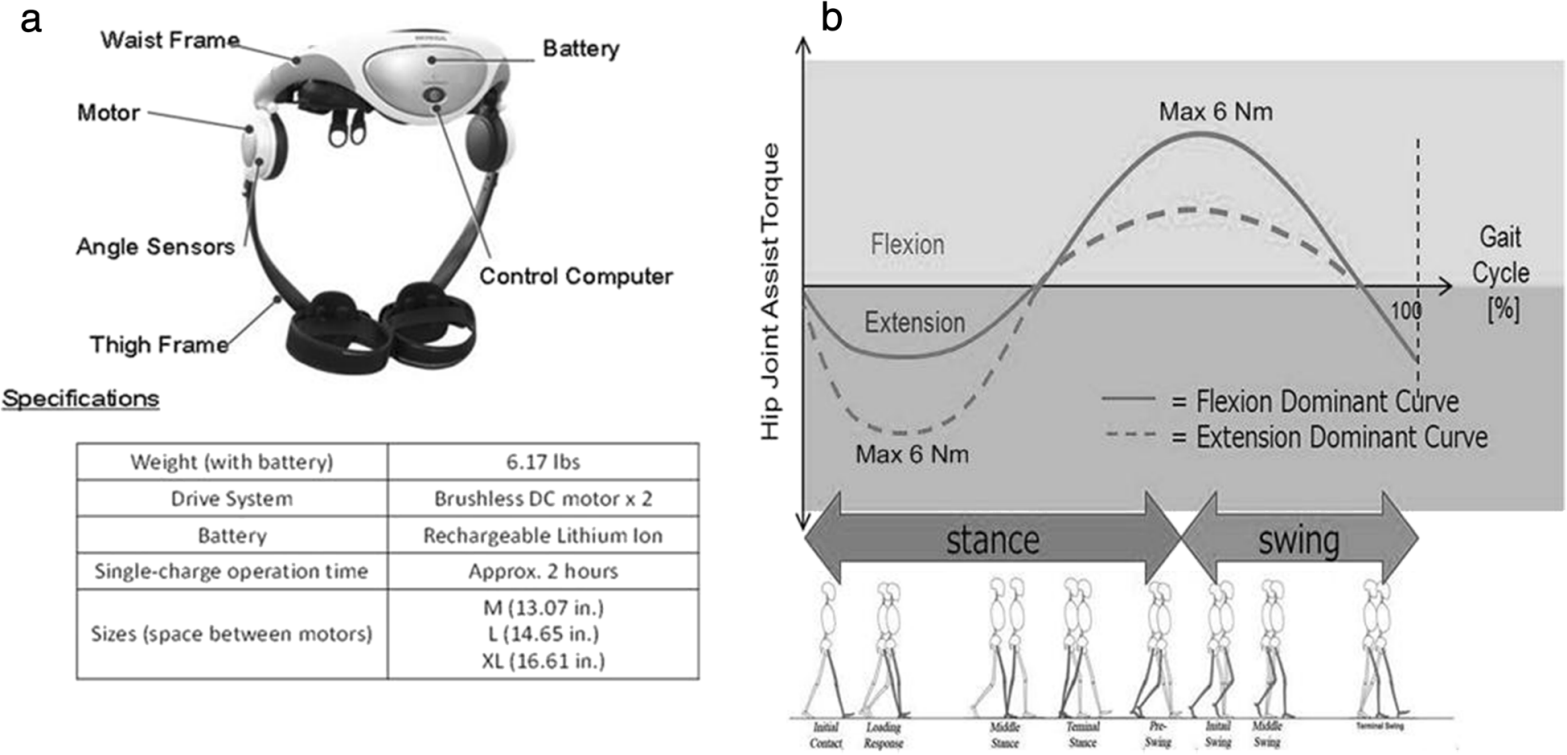
**a**. Honda Stride Management Assist (SMA®) Device **b**. Assist torque curve during gait cycle. Solid line indicates the changes in flexion assist torque and dotted line indicates changes in extension assist torque during gait cycle

The Stride Management Assist (SMA®) System is a new wearable robotic device developed by Honda R&D Corporation®, Japan (Fig. 1a). The SMA® was developed to enhance walking performance and to increase the community mobility and social interaction in elderly adults and patients with gait disorders [39–41]. The SMA® is worn around the hips and provides independent, active flexion and extension at each hip joint to assist the user during ambulation. However, there is limited evidence on the effect of such robotic exoskeletons on spatiotemporal gait characteristics, and very few studies have looked at the impact of a robotic exoskeleton on walking performance in the mild-moderate stroke population. In this study, we evaluate the effects of using the SMA® during task-specific training, compared to conventional physical therapy, in stroke survivors.

This study is part of a larger clinical trial, which aims to determine the impact of two different therapy approaches on various characteristics of gait, cortical drive to lower limb muscles, functional walking endurance, and functional balance in subjects with chronic stroke. The purpose of this study was to determine the effects of task-specific walking training with the SMA® device (SMA) vs. Functional Task Specific Training (FTST) on the spatiotemporal characteristics of gait in an outpatient setting for individuals post stroke. The task-specific walking training focused primarily on gait training with SMA; the FTST focused on addressing the individual patient’s functional goals, as planned with the physical therapists. In this study, all participants’ main functional goal was to improve gait function.

## Methods

### Trial design

**Fig. 2:**
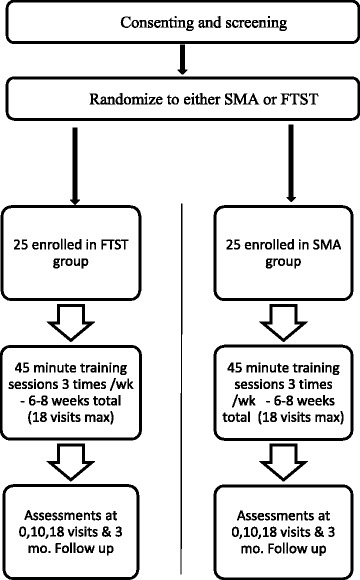
Study design schematic

This study was a randomized controlled trial comparing the effects of task-specific walking training with the SMA vs. FTST on spatiotemporal gait parameters. Subjects in both groups received training 3 times per week for 6–8 weeks, for a maximum of 18 training sessions. Each session was directed by a licensed physical therapist and lasted 45 min. Gait assessments were performed at visits 0 (baseline), 10 (mid-test) 18 (post-test), and at 3 months (follow-up) after training. Participants did not receive any other therapy sessions during the 3-month follow-up period (see Fig. 2 for study schematic).

### Participants

A total of 50 eligible subjects (33 male and 17 female) with chronic stroke (minimum time since stroke of one year) were recruited for the study and were randomized into either the SMA group (*n* = 25; 17 male and 8 female) or the FTST group (*n* = 25; 16 male and 9 female) using a random number generator (Table 1). Study inclusion criteria included being medically stable; an age between 18 and 85 years; an initial gait speed between 0.4 to 0.8 m/s (limited community ambulator); a score greater than 17 on the Mini-Mental State Examination (MMSE); an ability to sit unsupported for 30 s; an ability to walk at least 10 m with maximum 1 person assist; the ability to follow a 3-step command; and physician approval for patient participation. Exclusion criteria included serious cardiac conditions within the last 3 months; severe arthritis or orthopedic problems that limit lower extremity passive range of motion (knee flexion contracture of >10°, knee flexion ROM <90°, hip flexion contracture >25°, and ankle plantar flexion contracture of >15°); pre-existing neurological disorders such as Parkinson’s disease, Amyotrophic Lateral Sclerosis (ALS), Multiple Sclerosis (MS), dementia; history of major head trauma, lower extremity amputation, non-healing ulcers of a lower extremity, renal dialysis or end stage liver disease; legal blindness or severe visual impairment; pacemakers or metal implants in the head region; usage of medications that lower seizure thresholds; history of concussion in last 6 months; pregnant, nursing, or planning a pregnancy; participation in another clinical trial that, in the opinion of the Principal Investigator (PI), would likely affect the study outcome or confound the results. All subjects provided informed consent prior to participation in the study, which was approved by the Northwestern University Institutional Review Board. All study procedures were carried in accordance with the standards listed in Declaration of Helsinki, 1964.

**Table 1:** Demographic characteristics of all participants who completed the study

Patient Demographics	FTST (*n* = 25)	SMA (*n* =25)
Age (Years)	62 ± 3	60 ± 2
Gender (Male/ Female)	16/9	17/8
Hemiparesis (Right / Left)	12/13	13/12
Post Stroke (years)	5.4 ± 0.8	7.1 ± 1.5
Initial Gait speed (m/sec)	0.65 ± 0.02	0.7 ± 0.03

### Study settings

Subjects were recruited from the Chicago area. Based on their convenience and ability to commute, subjects were referred to one of the Rehabilitation Institute of Chicago (RIC) outpatient stroke rehabilitation clinics, either in downtown Chicago, or in the suburbs of Northbrook, IL, or Willowbrook, IL. Training sessions were completed by licensed RIC clinical physical therapists.

### Devices

The Stride Management Assist (SMA®) device is a robotic exoskeleton developed by Honda R&D Corporation®, Japan; (http://corporate.honda.com/innovation/walk-assist/) (Fig. 1a). This device provides independent assistance with hip flexion and extension for each leg to increase step length. The device weighs 2.8 kg, and has two brushless DC motors running on a rechargeable lithium ion battery. The SMA® actuators are equipped with angle and current sensors to monitor the range of motion (degrees) of the user’s hip joints and the torque (Nm) generated by the SMA®. Assist torque generated by the SMA® actuators is transmitted to the thighs via thigh frames. A physical therapist operates the device and can remotely change assist settings through software on a tablet while the user is using the SMA.

The SMA® control architecture uses a mutual rhythm scheme to influence the user’s walking patterns. Gait rhythms are believed to be controlled by Central Pattern Generator (CPGs)*–*neural networks that generate rhythmic patterns of output, independent of sensory feedback [42]. The SMA® control law uses neural oscillators in conjunction with the user’s CPG to synchronize itself with user input [43]. Angle sensors embedded in the SMA® actuators detect the user’s hip joint angles throughout the gait cycle. These angles are input to the SMA® controller, which calculates hip joint angle symmetry. The SMA® then generates assist torques at specific instances during the gait cycle to regulate these walking patterns.

Figure 1b shows the SMA® assist torque curve overlaid to the indicated key phases of the gait cycle [44]. Walking is initiated by the subject. After initial contact, the extensor torque initiates and reaches its peak just before mid-stance. The SMA® then switches to flexion assist during terminal stance. The flexor torque reaches its peak around initial swing. Finally, the SMA® switches to extension assist during terminal swing, and the cycle repeats. Peak torque values for flexion and extension ultimately depend on user input. While the SMA is capable of outputting a maximum of 6 Nm of assist torque, peak torque values are contingent upon user hip joint dynamics determined from the angle sensors. The SMA® automatically manipulates the walking motion to increase walk ratio (step length/cadence) providing torque assistance during hip flexion and extension movements when walking is initiated. For example, if the SMA® detects hip joint angle asymmetry, then the SMA® assist pattern follows a more flexion dominant curve (Fig. 1b) for the leg with shorter stride length, in an attempt to better support the user. Depending on user hip joint angles, the peak flexor torque may be less than 6 Nm. The user has total control on how fast they walk. The SMA® is designed to provide assistance only in the sagittal plane; however, it does not restrict movement in other directions.

The SMA® device is available in 3 sizes: medium, large, and X-large and is worn around the waist like a belt, with the motors placed near the hips and frames around the thighs. The device is simple to use in a clinical setting, is easily adjustable to accommodate the requirements of each subject, and only one functional upper limb is needed to put it on.

### Interventions

Initial clinical evaluations of all participants, performed by the physical therapist, included a general assessment of strength, flexibility, balance, sensation, endurance, transfers, and gait. All training sessions were 45 min long (in line with traditional physical therapy practice guidelines) and were split into 3 units of 15 min. Splitting the therapy sessions into units is standard practice in physical therapy clinics for the purposes of insurance billing. However, the transition between units was seamless, with rest breaks given as needed. General descriptions of the two training groups are provided below:

### Functional Task Specific Training (FTST)

FTST is a standard physical therapy training program conducted at all clinics at the Rehabilitation Institute of Chicago. The training program is based on the functional goals of the stroke patient/subject, and is planned in discussion with their physical therapist prior to beginning of the therapy program. In this study, all participants chose improvement in gait function as their functional goal. Each 45 min training session for the FTST group comprised 15 min (1unit) of high intensity over-ground walking training/ treadmill training at a rated perceived exertion between 12–16 on a Borg Rate of Perceived Exertion Scale (RPE, range 6–20) or 75 % of age-predicted maximum heart rate (HRmax), followed by 30 min (2 units) of functional goal-based mobility training (based on subject’s functional goals). As all the subjects in this group indicated improved gait function as their primary functional goal, the functional gait training included walking on varied surfaces, multi directional stepping, stair climbing, dual tasking, obstacles, and community mobility (walking outside the clinic in and around the community).

### Task-specific walking training using the SMA® (SMA)

The SMA group training sessions consisted of 30 min (2 units) dedicated to high intensity over-ground walking training with SMA® (RPE: 12–16 or 75 % of HRmax), and 15 min (1 unit) of dynamic functional gait training with the SMA® (walking on varied surfaces, multi directional stepping, stair climbing, dual tasking, obstacles, community mobility, etc.).

### Outcome measures and baseline tests

All participants were evaluated by a research physical therapist, who was blinded to the participant’s training group. Gait analysis assessments were performed at the beginning of the study (baseline), mid-point (mid), and end (post) of the training sessions, and at a follow-up assessment 3 months later. The GAITRite®, a system used to measure and analyze various spatiotemporal aspects of gait, comprises an electronic walkway with a pressure-sensitive mat (asensor-rich area 36” wide and 202” long, with a spatial resolution of 0.5”). The system records an individual’s footfalls as they walk over it, and calculates gait parameters. Data were recorded at a sampling rate of 120Hz. During data acquisition, participants were allowed to wear their regular footwear, use their assistive device if necessary, and received only stand-by assist from the physical therapist when needed, to avoid undue influence on subjects’ gait patterns. Participants began walking approximately five feet before the start of the walkway, and continued walking five feet beyond it, to ensure time and space for acceleration and deceleration. Each subject performed a total of 6 passes (3 passes at their normal self-selected pace and 3 passes at their fastest possible pace) on the walkway during each testing session. Footfalls recorded during each pass were visually checked for completeness and automatically processed to remove imprints from assistive devices such as a cane, walker, etc. Gait parameters were estimated for each pass separately and average values for self-selected and fast-pace velocity trials at each assessment point were used for further analysis.

For this study, gait velocity, cadence, step time, step length, stride length, swing time, stance time, and double support time were determined. These gait parameters were used to manually calculate spatiotemporal asymmetries. Spatial asymmetry was calculated by finding the ratio of right step length to left step length, while temporal asymmetry was calculated by finding the ratio of right to left swing time [21, 22, 45]. The larger value, whether right or left, was always placed in the numerator to produce a ratio greater than or equal to 1.0. A ratio of 1.0 would indicate perfect symmetry between the right and left legs. Bilateral gait parameters such as step time, step length, stride length, swing time, stance time, and double support time were further grouped into impaired and non-impaired sides for further analysis and to better understand the effects of training.

### Data and statistical analysis

All values are presented as mean ± standard error of mean (SEM), and the alpha value was set at *p* < 0.05 to indicate statistical significance, unless noted otherwise. The differences in baseline gait parameters between groups (FTST and SMA) were compared using analysis of variance. Two-way repeated measures ANOVA was used to compare changes from baseline to the different assessment points between the groups. Bonferroni post-hoc pairwise comparisons were made to determine the significance of differences, when applicable. Within each group, paired t-tests were used to compare data from different assessment points. Comparisons were made between the baseline values and mid, post, and follow-up time points and also between mid and post time points. Bonferroni corrections were applied to account for multiple comparisons (α = 0.0083). Sigmaplot 11.0 (Systat Software Inc., San Jose, CA, USA) was used to perform all statistical analyses.

## Results

A total of 54 subjects were consented for the study, and 50 completed all 18 training sessions and subsequent assessments. Four participants dropped out prior to starting the study due to transportation issues and scheduling conflicts. No adverse events were reported during the entire duration of the study.

### Comparison of baseline data between test groups

Analysis of variance tests were used to compare baseline data for all outcome measures between FTST and SMA groups. No statistical difference in baseline measures was observed between the FTST and SMA training groups.

### Comparison of effects of training between test groups during assessments

The average values for all gait parameters at baseline and the change from baseline at each subsequent testing point are presented in Table 2. In the SMA group, step length values on the impaired side during the self-selected walking speed trials were significantly longer, and spatial asymmetry during fast-walking speed trials was significantly lower than in the FTST group. No other significant differences between groups were observed during either self-selected velocity or fast-velocity trials.

**Table 2:** Spatiotemporal characteristics at baseline and changes from baseline at mid, post and follow up assessments

Gait Parameter	FTST			SMA			*p* value
	Mid	Post	3Mo. Follow up	Mid	Post	3Mo. Follow up	
Velocity (Cm/s)							
Self-Selected Velocity (SSV)- Baseline	64.61 ± 2.75			69.91 ± 3.03			0.205
Change from Baseline	5.17 ± 1.59	24.1 ± 5.07	10.25 ± 3.22	8.87 ± 2.59	17.41 ± 2.23	19.16 ± 4.37	
Fast Velocity (FV) - Baseline	88.74 ± 3.79			95.51 ± 4.25			0.243
Change from Baseline	12.14 ± 3.12	20.13 ± 3.37	18.28 ± 3.12	17.62 ± 3.13	27.80 ± 3.17	20.96 ± 4.45	
Cadence (Steps/min)							
SSV trial- Baseline	90.20 ± 2.81			88.42 ± 2.50			0.638
Change from Baseline	2.59 ± 1.41	7.71 ± 2.27	3.0 ± 2.03	4.18 ± 1.4	7.25 ± 1.19	−1.4 ± 6.22	
FV trial - Baseline	104.89 ± 3.28			104.99 ± 3.94			0.845
Change from Baseline	4.77 ± 2.22	8.89 ± 2.13	5.19 ± 2.57	8.94 + 1.84	13.17+ 2.47	11.75 + 2.52	
Step Time (sec)							
SSV trial - Impaired side Baseline	0.81 ± 0.03			0.82 ± 0.03			0.754
Change from Baseline	−0.02 ± 0.01	−0.06 ± 0.02	−0.04 ± 0.02	−0.04 ± 0.01	−0.08 ± 0.01	−0.05 ± 0.02	
SSV trial - Non impaired side Baseline	0.55 ± 0.02			0.56 ± 0.02			0.796
Change from Baseline	−0.01 ± 0.01	−0.04 ± 0.01	−0.01 ± 0.01	−0.02 ± 0.01	−0.04 ± 0.01	−0.02 ± 0.01	
FV trial - Impaired side Baseline	0.69 ± 0.02			0.68 ± 0.03			0.950
Change from Baseline	−0.03 ± 0.01	−0.06 ± 0.01	−0.05 ± 0.02	−0.05 ± 0.01	−0.08 ± 0.01	−0.07 ± 0.01	
FV trial - Non impaired side Baseline	0.49 ± 0.02			0.49 ± 0.02			0.869
Change from Baseline	−0.02 ± 0.01	−0.03 ± 0.01	−0.03 ± 0.01	−0.04 ± 0.01	−0.05 ± 0.01	−0.04 ± 0.01	
Stance Time (sec)							
SSV trial - Impaired side Baseline	0.87 ± 0.03			0.86 ± 0.03			0.801
Change from Baseline	−0.03 ± 0.02	−0.08 ± 0.02	−0.05 ± 0.02	−0.05 ± 0.02	−0.09 ± 0.01	−0.05 ± 0.02	
SSV trial - Non impaired side Baseline	1.03 ± 0.03			1.03 ± 0.03			0.975
Change from Baseline	−0.04 ± 0.02	−0.09 ± 0.02	−0.06 ± 0.02	−0.06 ± 0.02	−0.05 ± 0.02	−0.09 ± 0.02	
FV trial - Impaired side Baseline	0.72 ± 0.02			0.71 ± 0.02			0.415
Change from Baseline	−0.03 ± 0.02	−0.07 ± 0.01	−0.06 ± 0.02	−0.06 ± 0.01	−0.09 ± 0.01	−0.07 ± 0.01	
FV trial - Non impaired side Baseline	0.86 ± 0.03			0.84 ± 0.03			0.715
Change from Baseline	−0.04 ± 0.02	−0.08 ± 0.02	−0.07 ± 0.02	−0.08 ± 0.01	−0.11 ± 0.01	−0.08 ± 0.01	
Swing Time (sec)							
SSV trial - Impaired side Baseline	0.49 ± 0.02			0.52 ± 0.02			0.204
Change from Baseline	−0.002 ± 0.01	−0.02 ± 0.01	−0.003 ± 0.01	−0.016 ± 0.01	−0.026+ 0.01	−0.016+ 0.02	
SSV trial - Non impaired side Baseline	0.32 ± 0.01			0.35 ± 0.01			0.108
Change from Baseline	0.005 + 0.004	−0.004 + 0.01	0.011 ± 0.01	0.001 + 0.004	0.002 ± 0.01	0.013 ± 0.01	
FV trial - Impaired side Baseline	0.45 ± 0.02			0.47 ± 0.02			0.472
Change from Baseline	−0.01 ± 0.01	−0.02 ± 0.01	−0.10 ± 0.02	−0.03 ± 0.01	−0.04 ± 0.01	−0.13 ± 0.02	
FV trial - Non impaired side Baseline	0.31 ± 0.01			0.33 ± 0.01			0.135
Change from Baseline	−0.001 ± 0.01	−0.004 ± 0.01	0.03 ± 0.02	−0.01 ± 0.006	−0.01 ± 0.01	−0.01 ± 0.02	
Double Support Time (sec)							
SSV trial - Impaired side Baseline	0.55 ± 0.02			0.52 ± 0.02			0.337
Change from Baseline	−0.04 ± 0.01	−0.08 ± 0.02	−0.06 ± 0.02	−0.05 ± 0.01	−0.1 ± 0.01	−0.06 ± 0.01	
SSV trial - Non impaired side Baseline	0.55 ± 0.02			0.51 ± 0.02			0.288
Change from Baseline	−0.04 ± 0.01	−0.08 ± 0.02	−0.06 ± 0.02	−0.05 ± 0.01	−0.1 ± 0.01	−0.06 ± 0.01	
FV trial - Impaired side Baseline	0.41 ± 0.02			0.38 ± 0.02			0.234
Change from Baseline	−0.03 ± 0.01	−0.06 ± 0.01	−0.06 ± 0.01	−0.05 ± 0.01	−0.08 ± 0.01	−0.05 ± 0.01	
FV trial - Non impaired side Baseline	0.41 ± 0.02			0.38 ± 0.02			0.238
Change from Baseline	−0.03 ± 0.01	−0.06 ± 0.01	−0.06 ± 0.01	−0.05 ± 0.01	−0.08 ± 0.01	−0.05 ± 0.01	
Step Length (cm)							
SSV trial - Impaired side Baseline	48.09 ± 1.6			50.37 ± 2.19			0.411
Change from Baseline	2.41 ± 0.79	4.89 ± 1.19	3.55 ± 1.57	4.21 ± 1.08	8.55 ± 1.14	6.27 ± 1.14	
SSV trial - Non impaired side Baseline	38.36 ± 1.95			44.26 ± 2.13			0.051
Change from Baseline	2.28 ± 0.66	5.95 ± 1.26	6.43 ± 1.46	3.11 ± 0.86	6.54 ± 1.33	4.97 ± 1.76	
FV trial - Impaired side Baseline	55.98 ± 0.19			58.70 ± 2.43			0.389
Change from Baseline	4.07 ± 0.96	5.69 ± 1.30	5.68 ± 1.68	5.13 ± 0.94	7.96 ± 1.12	2.18 ± 3.93	
FV trial - Non impaired side Baseline	46.05 ± 2.10			51.92 ± 2.38			0.073
Change from Baseline	4.30 ± 1.07	7.29 ± 1.49	8.51 ± 1.83	4.12 ± 0.96	7.20 ± 1.28	3.66 ± 1.76	
Stride Length (cm)							
SSV trial - Impaired side Baseline	86.83 ± 2.93			94.76 ± 3.89			0.124
Change from Baseline	4.72 ± 1.21	10.61 ± 2.28	10.13 ± 2.95	8.14 ± 1.94	15.31 ± 2.15	11.67 ± 2.59	
SSV trial - Non impaired side Baseline	86.80 ± 2.89			95.01 ± 3.88			0.135
Change from Baseline	4.72 ± 1.13	10.79 ± 2.27	10.02 ± 2.89	7.18 ± 1.78	14.93 ± 2.12	11.42 ± 2.67	
FV trial - Impaired side Baseline	102.49 ± 3.14			110.86 ± 4.47			0.124
Change from Baseline	8.24 ± 1.77	12.9 ± 2.48	13.93 ± 3.21	9.47 ± 1.78	15.17 ± 2.22	9.997 ± 2.67	
FV trial - Non impaired side Baseline	102.42 ± 3.14			111.09 ± 4.46			0.129
Change from Baseline	8.35 ± 1.80	12.75 ± 2.38	14.10 ± 3.22	8.98 ± 1.57	15.06 ± 2.09	9.85 ± 2.81	
Spatial Asymmetry							
SSV trial- Baseline	1.37 ± 0.08			1.25 ± 0.05			0.204
Change from Baseline	−0.03 ± 0.05	−0.08 ± 0.04	−0.07 ± 0.04	0.002 ± 0.02	−0.01 ± 0.04	−0.06 ± 0.04	
FV trial - Baseline	1.33 ± 0.07			1.19 ± 0.05			0.053
Change from Baseline	−0.07 ± 0.03	−0.1 ± 0.04	−0.09 ± 0.04	−0.002 ± 0.02	−0.01 ± 0.03	0.04 ± 0.02	
Temporal Asymmetry							
SSV trial- Baseline	1.52 ± 0.05			1.50 ± 0.04			0.758
Change from Baseline	−0.03 ± 0.02	−0.04 ± 0.03	−0.06 ± 0.02	−0.04 ± 0.02	−0.08 ± 0.02	−0.08 ± 0.04	
FV trial - Baseline	1.44 ± 0.04			1.41 ± 0.04			0.654
Change from Baseline	−0.02 ± 0.03	−0.05 ± 0.03	−0.04 ± 0.03	−0.05 ± 0.02	−0.08 ± 0.03	−0.04 ± 0.03	

### Effects of SMA and FTST training on gait parameters

Both the SMA and FTST training groups showed significant within-group improvements in numerous gait parameters, which are indicated in Tables 3 and 4, where ‘Yes’ indicates significant improvements and ‘No’ indicates no significant change.

**Table 3:** Within-group comparisons of spatiotemporal characteristics during self-selected velocity (SSV) walking trials: pre = baseline; Mid = Mid-training test; Post = post-training test; Follow = follow-up test

Gait parameter	Mid Vs. Pre	Post Vs. Pre	Follow Vs. Pre	Post Vs. Mid	Follow Vs. Mid	Follow Vs. Post
SSV Velocity -FTST	Yes	Yes	Yes	No	No	No
SSV Velocity - SMA	Yes	Yes	Yes	Yes	No	No
SSV Cadence - FTST	No	Yes	No	No	No	No
SSV Cadence - SMA	Yes	Yes	No	No	No	No
SSV Step Time - Impaired - FTST	No	Yes	No	No	No	No
SSV Step Time - Impaired - SMA	No	Yes	No	No	No	No
SSV StepTime- Non Impaired - FTST	No	Yes	No	No	No	No
SSV StepTime- Non Impaired - SMA	Yes	Yes	No	No	No	No
Step Length- Impaired - FTST	Yes	Yes	No	No	No	No
Step Length- Impaired - SMA	Yes	Yes	Yes	Yes	No	No
Step Length - Non Impaired- FTST	Yes	Yes	Yes	No	Yes	No
Step Length - Non Impaired- SMA	Yes	Yes	No	No	No	No
Stride Length - Impaired - FTST	Yes	Yes	Yes	No	No	No
Stride Length - Impaired - SMA	Yes	Yes	Yes	Yes	No	No
Stride Length - Non Impaired - FTST	Yes	Yes	Yes	No	No	No
Stride Length - Non Impaired - SMA	Yes	Yes	Yes	Yes	No	No
SSV Swing Time - Impaired - FTST	No	No	No	No	No	No
SSV Swing Time - Impaired - SMA	No	Yes	No	No	No	No
SSV Swing Time - Non Impaired - FTST	No	No	No	No	No	No
SSV Swing Time - Non Impaired - SMA	No	No	No	No	No	No
SSV Stance Time - Impaired- FTST	No	Yes	No	No	No	No
SSV Stance Time - Impaired- SMA	Yes	Yes	Yes	No	No	No
SSV Stance time - Non Impaired- FTST	No	Yes	No	No	No	No
SSV Stance time - Non Impaired- SMA	Yes	Yes	Yes	Yes	No	No
SSV Double Supp. Time- Impaired - FTST	No	Yes	No	No	No	No
SSV Double Supp. Time- Impaired - SMA	Yes	Yes	Yes	Yes	No	No
SSV Double Supp. Time- Non Impaired - FTST	No	Yes	No	No	No	No
SSV Double Supp. Time- Non Impaired - SMA	Yes	Yes	Yes	Yes	No	No
SSV Spatial Asymmetry - FTST	No	No	No	No	No	No
SSV Spatial Asymmetry - SMA	No	No	No	No	No	No
SSV Temporal Asymmetry - FTST	No	No	No	No	No	No
SSV Temporal Asymmetry - SMA	No	Yes	No	No	No	No

**Table 4:** Within-group comparisons of spatiotemporal characteristics during fast velocity (FV) walking trials: Pre = baseline; Mid = mid-training test; Post = post-training test; Follow = follow-up test

Gait parameter	Mid Vs. Pre	Post Vs. Pre	Follow Vs. Pre	Post Vs. Mid	Follow Vs. Mid	Follow Vs. post
FV velocity - FTST	Yes	Yes	Yes	Yes	No	No
FV velocity - SMA	Yes	Yes	Yes	Yes	No	No
FV Cadence - FTST	No	Yes	No	Yes	No	No
FV Cadence - SMA	Yes	Yes	Yes	No	No	No
FV Step Time- Impaired - FTST	No	Yes	No	Yes	No	No
FV Step Time- Impaired- SMA	Yes	Yes	Yes	No	No	No
FV Step Time - Non Impaired- FTST	No	Yes	No	No	No	No
FV Step Time - Non Impaired- SMA	Yes	Yes	Yes	No	No	No
FV Step Length - Impaired- FTST	Yes	Yes	Yes	No	No	No
FV Step Length - Impaired- SMA	Yes	Yes	Yes	Yes	No	No
FV Step Length - Non impaired- FTST	Yes	Yes	Yes	Yes	Yes	No
FV Step Length - Non impaired- SMA	Yes	Yes	No	Yes	No	No
FV Stride Length - Impaired - FTST	Yes	Yes	Yes	No	Yes	No
FV Stride Length - Impaired- SMA	Yes	Yes	Yes	Yes	No	No
FV Stride Length - Non Impaired- FTST	Yes	Yes	Yes	No	Yes	No
FV Stride Length - Non Impaired- SMA	Yes	Yes	Yes	Yes	No	No
FV Swing time - Impaired- FTST	No	No	Yes	No	Yes	Yes
FV Swing time - Impaired- SMA	Yes	Yes	Yes	No	Yes	Yes
FV Swing time - Non Impaired-FTST	No	No	No	No	No	No
FV Swing time - Non Impaired-SMA	No	No	No	No	No	No
FV Stance Time - Impaired- FTST	No	Yes	Yes	No	No	No
FV Stance Time - Impaired- SMA	Yes	Yes	Yes	No	No	No
FV Stance Time - Non Impaired- FTST	No	Yes	Yes	Yes	No	No
FV Stance Time - Non Impaired-SMA	Yes	Yes	Yes	No	No	No
FV Double Supp. Time - Impaired- FTST	No	Yes	Yes	No	No	No
FV Double Supp. Time - Impaired- SMA	Yes	Yes	Yes	Yes	No	Yes
FV Double Supp. Time - Non Impaired-FTST	No	Yes	Yes	No	No	No
FV Double Supp. Time - Non Impaired-SMA	Yes	Yes	Yes	Yes	No	No
FV Spatial Asymmetry -FTST	No	No	No	No	No	No
FV Spatial Asymmetry -SMA	No	No	No	No	No	No
FV Temporal Asymmetry- FTST	No	No	No	No	No	No
FV Temporal Asymmetry- SMA	No	Yes	No	No	No	No

However, within the SMA group, significant improvements in additional spatiotemporal variables were observed compared to the FTST group. Those additional improvements are discussed below, in comparison to results from the FTST group.

### Gait velocity

In self-selected walking velocity trials, significantly improved gait speeds were achieved in both groups. Both groups had statistically significant increases in walking speed at mid-, post- and follow-up testing compared to baseline values. However, in addition, in the SMA group, significant improvements were also observed between mid- and post-test walking speed velocity (*p* <0.008).

In fast-velocity walking trials, both groups showed significant increases in gait velocity at mid-, post-, and follow-up testing compared to baseline and between the mid- and post-testing time points (*p* <0.008).

### Cadence

During self-selected walking speed trials, a significant increase in cadence was observed only at post-test compared to baseline (*p* < 0.008) in the FTST group. However, in the SMA group, significant changes were also seen at mid- and post-test compared to baseline (*p* <0.008).

For fast-walking trials, the FTST group had a significant increase in cadence at post-test compared to baseline (*p* < 0.008) and between post- and mid-test (*p* <0.008). In the SMA group, cadence at mid-, post- and follow-up testing was also significantly increased over baseline (*p* <0.008).

### Step time

In self-selected walking velocity trials, step times were significantly lower at post-test compared to baseline on the impaired side in both the training groups (*p* < 0.008). On the non-impaired side, for the FTST group, step times were significantly lower at the post-test when compared to baseline (*p* <0.008). However, non-impaired step times were significantly lower at both mid- and post-tests compared to baseline only in the SMA group (*p* <0.008).

In fast-velocity walking trials, the FTST group showed significantly lower step times at post-test compared to baseline and mid-test for the impaired side (*p* <0.008), and on the non-impaired side, post-test values were lower than baseline (*p* < 0.008). However, the SMA group had significantly lower step times at mid-, post-, and follow-up testing compared to baseline in both impaired and non-impaired sides (*p* <0.008).

### Stance time

In self-selected walking speed trials, the FTST group showed significant reduction in stance time on both the impaired and non-impaired sides at post-test compared to baseline (*p* < 0.008). However, for the SMA group, a decreased stance time was observed at mid-, post- and follow-up testing on both the impaired and non-impaired sides (*p* <0.008). Furthermore, a significant decrease was identified between mid- and post-test stance times on the non-impaired side (*p* <0.008).

During fast-velocity walking trials, the FTST group had significantly shorter stance times at post- and follow-up testing compared to baseline on both the impaired and non-impaired side. In addition, the non-impaired side also had a significant decrease at post- compared to mid-test values (*p* < 0.008). However, in the SMA group, stance time decreased significantly at mid-, post- and follow-up testing compared to baseline on both the impaired and non-impaired sides (*p* < 0.008).

### Swing time

In self-selected walking speed trials, swing time decreased significantly on the impaired side at post-test compared to baseline value in the SMA group (*p* <0.008), while no significant changes were observed in the FTST group.

In fast-velocity walking trials, swing time was significantly decreased at follow-up compared to baseline on the impaired side in the FTST groups (*p* < 0.008). In contrast, in the SMA group, significant decreases were observed at mid-, post- and follow-up tests on the impaired side (*p* < 0.008). Swing times at follow-up on the impaired side were significantly lower compared to mid- and post-test values in both groups (*p* < 0.008). No changes were observed on non-impaired sides in either group.

### Double support time

During self-selected walking speed trials, both training groups had a significant decrease in double support time at post-test compared to baseline in both the impaired and non-impaired sides (*p* < 0.008). Additionally, the SMA group had significantly lower values at mid- and follow-up tests compared to baseline (*p* < 0.008) and a significant decrease between mid- and post-test in both the impaired and non-impaired side (*p* < 0.008).

In fast-velocity walking trials, both training groups showed a significant decrease in double support time at post- and follow-up testing compared to baseline values for both impaired and non-impaired sides (*p* <0.008). Additionally, in the SMA group, a significant decrease was also found at mid-test compared to baseline, and the decreases between mid- to post-test (both sides) and post-test to follow up (impaired side) were significant (*p* < 0.008).

### Step length

During self-selected walking speed trials, subjects showed a significant increase in step length at mid- and post-tests compared to baseline on the impaired side and non-impaired sides in both training groups (*p* < 0.008). Additionally, in the SMA group, a significant increase in step length was also found at follow up vs. baseline and post vs. mid time points on the impaired side. The non-impaired side had significant increases at follow-up when compared to both pre- and mid- values in the FTST group. (*p* < 0.008)

In fast-pace walking trials, the impaired side in both groups showed an increase in step length at mid-, post-, and follow-up tests from baseline level (*p* < 0.008). In addition, in the SMA group, impaired-side step length increased significantly from mid- to post-test (*p* < 0.008). On the non-impaired side, an increase in step length was observed at mid-and post-tests compared to baseline in both training groups. Additionally, the FTST group showed significant increases in step length between baseline and follow-up, and mid-time points vs. post and follow-up.

### Stride length

During the self-selected walking speed trials, impaired and non-impaired sides showed a significant increase in stride length at mid-, post- and follow-up testing points compared to baseline in both the FTST and SMA groups (*p* < 0.008). In addition, in the SMA group, the increase between mid- to post- was also significant on both sides (*p* < 0.008)

Similar results were observed in fast-pace walking trials, where both impaired and non-impaired sides showed a significant increase in stride length at mid-, post- and follow-up compared to baseline. Stride-length in the FTST group also increased significantly from mid- to follow-up values for both the impaired and non-impaired sides. In the SMA group the increase observed from mid- to post- was significant on both sides (*p* < 0.008).

### Spatial asymmetry

Although there were statistically significant differences between groups, no statistically significant changes in spatial asymmetry values were found within groups or between sides (impaired vs. non-impaired), either during self-selected or fast walking velocity trials.

### Temporal asymmetry

Within the SMA group, a significant decrease in temporal asymmetry was observed at post-testing compared to baseline, for both self-selected and fast walking velocity trials (*p* < 0.008). No significant decrease in temporal asymmetry was observed within the FTST group.

## Discussion

The results of this study show that short-term high-intensity training using either a light-weight wearable robot such as the SMA® or functional task-specific training can significantly impact spatiotemporal gait parameters in individuals with chronic gait impairments due to stroke. The only significant differences between training groups were an increased step length on the impaired side and reduction in spatial asymmetry within the SMA training group. However, the change in spatial asymmetry is more of a statistical change than a true clinical change. For both groups, intensive training (3 times/week) across groups over 6 weeks resulted in significant improvements in numerous spatiotemporal parameters of walking, specifically in: velocity, cadence, step time, stance time, swing time, double support time, stride length, and spatial asymmetry. This potentially demonstrates that both interventions are beneficial for stroke rehabilitation. However, the SMA® positively impacted more gait variables measured at multiple time points, showing that this device has promise as an appropriate and effective therapeutic wearable robotic device for outpatient rehabilitation. In addition, the SMA® is safe and poses no risk to the user.

Using over-ground light-weight wearable robots to target gait abnormalities is a relatively new concept, and clinical research in this area is quite limited. Our results are in line with the previous three studies on the SMA® conducted in young adults and in the elderly, where use of the SMA® resulted in positive changes in gait performance. In the previous studies the changes in gait performance were partially mediated by improvements in muscle activation, glucose metabolism, and improved energy efficiency during use of the SMA® [39, 40, 46]. Interestingly, several large studies using treadmill-based robotic technology have found that traditional physical therapy was a more effective intervention for improving gait function following a stroke than robotic technology [18, 47]. Results from our 50-subject study indicate that wearable robots can potentially provide improvements in gait function that are superior or equal to high-intensity traditional physical therapy. This finding may open up a field of research on the therapeutic effects of over-ground robots, which needs more extensive investigation.

One of the important variables we quantified in this study was gait speed, an important ambulation parameter that is continually addressed after a stroke, as improvements are known to directly impact quality of life in stroke survivors [48]. Minimal clinically important difference (MCID) for gait speed in the stroke population ranges from 10 cm/s [49] to 16 cm/s [50]. MCIDs are patient-derived scores that, following a clinical intervention, reflect the minimum changes that are meaningful for the patient. MCIDs are used in research and clinical practice to make decisions on the therapeutic gains made by the patient. Both the SMA and FTST groups in this study showed increased gait speed above the established MCID for stroke survivors in both post- (SMA: fast velocity = 27.80 cm/s, self-selected velocity = 17.41 cm/s; FTST: fast-velocity = 20.13 cm/s, Self-selected velocity = 24.1 cm/s) and 3-month follow up trials (SMA: Fast Velocity = 20.96 cm/s, self-selected velocity = 19.16 cm/s; FTST: fast velocity = 18.28 cm/s, self-selected velocity = 10.25 cm/s). Gait speed in our study increased slightly more than has previously been described [18]. Thus, both training interventions, when performed at high intensity and dosage can have significant effects on gait within a short period of time (six weeks). In addition, the SMA® may provide clinicians with the ability to continue physical rehabilitation at home, as a take-home mobility device.

One possible explanation for the effect of SMA® training on gait speed is that gait speed after stroke is found to be impacted by the paretic side hip flexors, which often compensate for plantar-flexor impairment following stroke [51]. During late stance, the hip flexors pull the leg upwards and forwards, advancing the leg further before the subsequent heel strike, which contributes to swing initiation [51]. Therefore it is possible that stroke subjects with variable stepping on the paretic side have reduced paretic leg advancement during swing due to impaired paretic leg hip flexor activity in pre-swing. Therefore, an intervention that corrects for this abnormality might help the hip-flexors in pre-swing and subsequently help the leg to advance in swing phase. This may be valuable in stroke rehabilitation and lead to improvements in gait. In the current study, the hip flexor/extensor assist provided by the SMA® device may be a more effective intervention than standard physical therapy training in targeting hip flexor weakness on the paretic side.

To understand the impact of the SMA® on gait function we studied many other spatiotemporal parameters impacted by stroke. Following a stroke, individuals have a characteristic gait pattern that shows variable step/stride length (shorter or longer) on the paretic side compared to the non-paretic side, and a relatively variable swing phase (longer or shorter) on the paretic side compared to the non-paretic side [52]. This increased variability in spatial and temporal variables lead to poor dynamic balance and a decline in gait speed and function.

In both the FTST and SMA groups, statistically significant within-group changes in several parameters (including cadence, swing time, double support time, and stride length) with positive influences on gait performance were seen across time. Cadence increased, and an increase in the cadence of individuals post-stroke is thought to demonstrate an improved gait performance [53, 54]. Swing time was found to decrease on the impaired side in both the FTST and SMA groups. One typical characteristic of asymmetry seen in individuals post-stroke is a variable swing time of the paretic limb compared on the non-paretic limb (i.e., temporal asymmetry) [12–14]. As a result, a decrease in swing time on the impaired side could indicate a trend towards improved temporal asymmetry. Double support time was found to decrease in both groups. This too is considered an advantageous change in gait because increased double support time has been shown to lead to difficulty with balance and decreased energy efficiency during ambulation [55]. Temporal asymmetry in the SMA training group decreased in both fast and self-selected velocity speeds. However, the observed change of 0.08 in temporal symmetry for both walking speeds does not seem to reach the MDC values published in a manuscript by Lewek et al. [56]. Interestingly, a greater number of spatiotemporal variables improved in the SMA group over time than in the FTST group; however, it is difficult to make any conclusions on whether the SMA group is significantly better than FTST based on these study data. Analysis of the clinical, physiological, and community stepping data from the larger data set of the full, ongoing clinical trial will give us a better insight. Overall, our study indicates that high-intensity training over just 18 sessions in both the FTST and SMA groups improved spatiotemporal gait parameters in individuals with stroke, with a trend towards a more symmetrical and efficient gait pattern. Bringing individuals closer to a symmetrical gait pattern could impact energy efficiency, gait speed, and balance control, and decrease the risk of falls, lower extremity musculoskeletal injury, and improve overall quality of gait in the stroke population [22].

Some of the differences in performance noted between the SMA and the FTST groups likely result from differences in the way each intervention targeted the abnormal gait parameters. The SMA device functions by generating assistance in active hip flexion and extension for each side independently. This group’s intervention consisted of mainly high-intensity over-ground gait training with some functional training. The FTST group received no direct external robotic assistance with ambulation; however, they additionally received high-intensity treadmill training, combined with over-ground gait and functional training.

### Limitations

This study has a number of limitations including length of study, and the SMA® device fit. The intervention was limited to 6–8 weeks with a follow-up period of 3 months. The majority of timing effects were seen at mid- to post-tests, indicating that an intervention needed to take place for at least 6 weeks to be effective. Determining whether the effects of these interventions persist for longer than 3 months was beyond the scope of this study, further research to determine the ideal length of therapy to achieve long-lasting therapeutic effects would be beneficial. Another limitation exists in the fit accuracy of the SMA device to each individual patient. Only standard sizes of the SMA device were used, i.e., medium, large or extra-large. As for any orthotic, one size does not fit everyone, and a more customized fit might have further enhanced outcomes in the SMA users.

## Conclusions

In conclusion, a short time period (6 weeks) with 18 therapy sessions for both the SMA and FTST interventions provided similar, significant improvements in a majority of spatiotemporal gait parameters, including velocity, cadence, step time, stance time, swing time, double support time, stride length, and spatial asymmetry. However, the SMA® device was more effective at improving additional spatiotemporal parameters across different time points. Improvements in these gait parameters can have a positive effect on functional mobility and quality of life in stroke survivors. The wearable over-ground robotic SMA® device proved to be appropriate for gait training, safe, easy to use, and posed no risk to users, indicating that it could be safely implemented in a home setting. Further research is needed to determine the importance of intervention length and long-term effects, as well as the feasibility of using this device in a clinic versus a home setting.

## Abbreviations

(FTST): Functional task specific training
(SMA): Stride Management Assist
(FSR): Force Sensitive Resistor
(MMSE): Mini-Mental State Examination
(SEM): Standard Error of Mean
(MCID): Minimal clinically important difference
